# Implementing Exercise = Medicine in routine clinical care; needs for an online tool and key decisions for implementation of Exercise = Medicine within two Dutch academic hospitals

**DOI:** 10.1186/s12911-022-01993-5

**Published:** 2022-09-22

**Authors:** Adrie Bouma, Femke van Nassau, Joske Nauta, Leonie Krops, Hidde van der Ploeg, Evert Verhagen, Lucas van der Woude, Helco van Keeken, Rienk Dekker, PIE = M consortium, Willem van Mechelen, Vincent de Groot, Marike van der Leeden, Johannes Zwerver, Martin Fluit, Inge van den Akker-Scheek, Martin Stevens, Ronald Diercks, Willem Bossers, Laurien Buffart, Johan de Jong, Caroline Kampshoff, Hans Leutscher, Sacha van Twillert

**Affiliations:** 1 Department of Rehabilitation Medicine, University Medical Centre Groningen, University of Groningen, Groningen, The Netherlands; 2 Department of Public and Occupational Health, Amsterdam University Medical Centres, Amsterdam Public Health Research Institute, Amsterdam, The Netherlands; 3 Centre for Human Movement Sciences, University Medical Centre Groningen, University of Groningen, Groningen, The Netherlands; 4 Department of Rehabilitation Medicine, Amsterdam Movement Sciences, Vrije Universiteit Amsterdam, Amsterdam, The Netherlands; 5 Sports Valley, Sports Medicine, Gelderse Vallei Hospital, Ede, The Netherlands; 6 Stichting Special Heroes Nederland, Arnhem, The Netherlands; 7 Department of Orthopedic Surgery, University Medical Center Groningen, University of Groningen, Groningen, Netherlands; 8 The Lifelines Cohort Study, Roden, The Netherlands; 9 Department of Physiology, Radboud University Medical Center, Nijmegen, The Netherlands; 10 Research Group Applied Sports Science, School of Sports Studies, Hanze University of Applied Sciences Groningen, Groningen, The Netherlands; 11 Department of Medical Oncology, Cancer Center Amsterdam, Amsterdam University Medical Centers, Vrije Universiteit Amsterdam, Amsterdam, The Netherlands; 12 Knowledge Centre for Sport and Physical Activity, Ede, The Netherlands; 13 Center of Expertise on Quality and Safety, University Medical Center Groningen, University of Groningen, Groningen, The Netherlands

**Keywords:** Exercise is Medicine, Physical activity, Lifestyle, Prescription, Advice, Clinician, Referral, Decision-making, Tool, Digital health

## Abstract

**Background:**

There is much evidence to implement physical activity interventions for medical reasons in healthcare settings. However, the prescription of physical activity as a treatment, referring to as ‘Exercise is Medicine’ (E = M) is currently mostly absent in routine hospital care in The Netherlands. To support E = M prescription by clinicians in hospitals, this study aimed: (1) to develop an E = M-tool for physical activity advice and referrals to facilitate the E = M prescription in hospital settings; and (2) to provide an E = M decision guide on key decisions for implementation to prepare for E = M prescription in hospital care.

**Methods:**

A mixed method design was used employing a questionnaire and face-to-face interviews with clinicians, lifestyle coaches and hospital managers, a patient panel and stakeholders to assess the needs regarding an E = M-tool and key decisions for implementation of E = M. Based on the needs assessment, a digital E = M-tool was developed. The key decisions informed the development of an E = M decision guide.

**Results:**

An online supportive tool for E = M was developed for two academic hospitals. Based on the needs assessment, linked to the different patients’ electronic medical records and tailored to the two local settings (University Medical Center Groningen, Amsterdam University Medical Centers). The E = M-tool existed of a tool algorithm, including patient characteristics assessed with a digital questionnaire (age, gender, PA, BMI, medical diagnosis, motivation to change physical activity and preference to discuss physical activity with their doctor) set against norm values. The digital E = M-tool provided an individual E = M-prescription for patients and referral options to local PA interventions in- and outside the hospital. An E = M decision guide was developed to support the implementation of E = M prescription in hospital care.

**Conclusions:**

This study provided insight into E = M-tool development and the E = M decision-making to support E = M prescription and facilitate tailoring towards local E = M treatment options, using strong stakeholder participation. Outcomes may serve as an example for other decision support guides and interventions aimed at E = M implementation.

**Supplementary Information:**

The online version contains supplementary material available at 10.1186/s12911-022-01993-5.

## Background

Insufficient physical activity (PA) is a substantial public health problem [1]. Insufficient PA is a leading risk factor for major non-communicable diseases and has a negative effect on mental health and quality of life [2]. Inactivity is defined as not doing at least 150 min of moderate-intensity, or 75 min of vigorous-intensity physical activity per week, or any equivalent combination of the two and includes physical activity at work, at home, for commuting, and during leisure time [3]. The health benefits of a physically active lifestyle are numerous [4]. These not only apply for healthy individuals, but may be even more important for those with serious (chronic) health conditions. The benefits of providing PA advice and/or exercise in a clinical setting are multiple. They include positive effects on physical factors, psychological factors and quality of life [5, 6] and a decrease in postoperative complications, following exercise-based pre-habilitation [7]. Despite the beneficial effects of PA, PA levels in people with chronic health conditions are low [8–10]. In the Dutch general population 52% of people between 18 and 65 years old meet the PA guidelines and 40% of people over 65 years [11]. Compared to other countries, the Dutch are more active, although still a substantial group (48%) is insufficiently active [12].

The WHO has included specific advice for adults living with chronic conditions and those living with a disability into their PA guidelines [3]. The WHO recommends that adults and older adults living with chronic conditions or disabilities should undertake regular PA, based on the same WHO guidelines. The American College of Sports Medicine (ACSM) uses the paradigm ‘Exercise is Medicine’ (EIM) in its global initiative to increase awareness to consider physical activity as a treatment option [13]. This EIM initiative has three primary aims: (1) to encourage healthcare providers to evaluate their patient’s PA level at each clinic visit, (2) to compare each patient’s current PA level with the national PA guidelines (based on the WHO guidelines), and (3) to provide PA counseling and/or referrals to each patient who does not meet the national PA guidelines [13, 14].

The WHO stated that professionals in healthcare settings can contribute substantially to counter this global inactivity pandemic by promoting PA [15, 16]. Previous research has shown that EIM interventions in healthcare settings increased levels of PA [17, 18] and encouraged healthcare practitioners to regularly assess and counsel patients on PA [19, 20]. This includes approaches for routinely integrating PA assessment, counseling and prescription or referral programs, particularly for patients with chronic diseases [18]. As such, PA prescription is proposed as an important component of standard medical care [21–25].

However, there is a lack of PA prescription and referrals from clinicians [18]. Barriers to the implementation and uptake of exercise prescription include; lack of time, insufficient skills to address patients’ PA behavior, insufficient knowledge of PA guidelines and referral options and subjective influences as immediate and significant barrier to this referral process [26–31]. Strategies to overcome these barriers should focus on increasing clinicians EIM referral skills, improving clinicians knowledge of EIM referral options and develop a support system to ensure that EIM is high on the priority list of clinicians [28].

Additionally, in a review of Stout et al. [32] a framework is proposed for efficient and effective screening in oncology clinical practice on five domains that enables exercise referrals best suited to an individual’s existing and evolving needs. They recommended to develop technical tools and systems to enhance healthcare professionals’ ability to engage patients around exercise and physical activity recommendations. A clinical decision aid includes prompts for the clinician to take them through the PA advice and referral steps [33]. In the EIM website The Physical Activity Vital Sign is recommended to support clinical screening [13]. They also recommend embedding PA assessments in the Electronic Medical Record (EMR) to increase the implementation of EIM in hospital care.

Because, there is more experience in the U.S.A with EIM, we used the knowledge about EIM to translate to Dutch hospital contexts, where EIM is not a component of standard medical care. In the Netherlands there is no shared decision-making in mapping inactivity and prescribing and referring inactive patients to PA interventions. Specific considerations for implementation are related to the development of a supportive EIM-tool and the attunement of implementation to the work process of clinical practices.

The ACSM uses the abbreviation EIM for ‘Exercise is Medicine’. However, many other terms have been used [34]. In this study, we used the abbreviation E = M, since the current study is part of a larger project, called Physicians Implement Exercise = Medicine (PIE = M), in which the abbreviation E = M is used [35]. PIE = M consists of three interrelated work packages. Firstly, the current implementation status, facilitators and barriers of E = M were investigated among clinicians from academic hospitals [28]. One of the recommended strategies to overcome barriers was to develop an E = M-tool. Therefore, in work package two, an E = M tool will be developed and an insight will be provided in decision-making to facilitate the implementation of E = M in clinical practice. Thirdly, we will pilot-implement the set of implementation strategies, including the E = M tool, to test its feasibility in routine care of clinicians in these two academic hospitals.

The aim of this study is to create an E = M tool for a patient PA advice for outpatient clinical setting, which in turn is customized to the needs and wishes of hospital departments Rehabilitation Medicine and Orthopedics. Additionally, the aim of this study is to provide insight into key decision to facilitate the implementation of E = M in hospital care, resulting in a guide to E = M decision-making. This study is conducted in the departments of Rehabilitation Medicine and Orthopedics. This was a pragmatic choice, since in these departments there was the energy and motivation to innovate clinical practice with PA on prescription. We started to pioneer with these front runners, while they understand exercise and patients functional-related needs. We will use outcomes as an example for scaling up to other departments.

## Methods

The current study is part of a larger PIE = M project, whose study protocol has been published elsewhere [35]. The PIE = M study was approved by The Human Research Ethics Committee of the University Medical Center Groningen (METc2017/517) and the University Medical Centers of Amsterdam (2018.219).

### Design

**Fig. 1:**
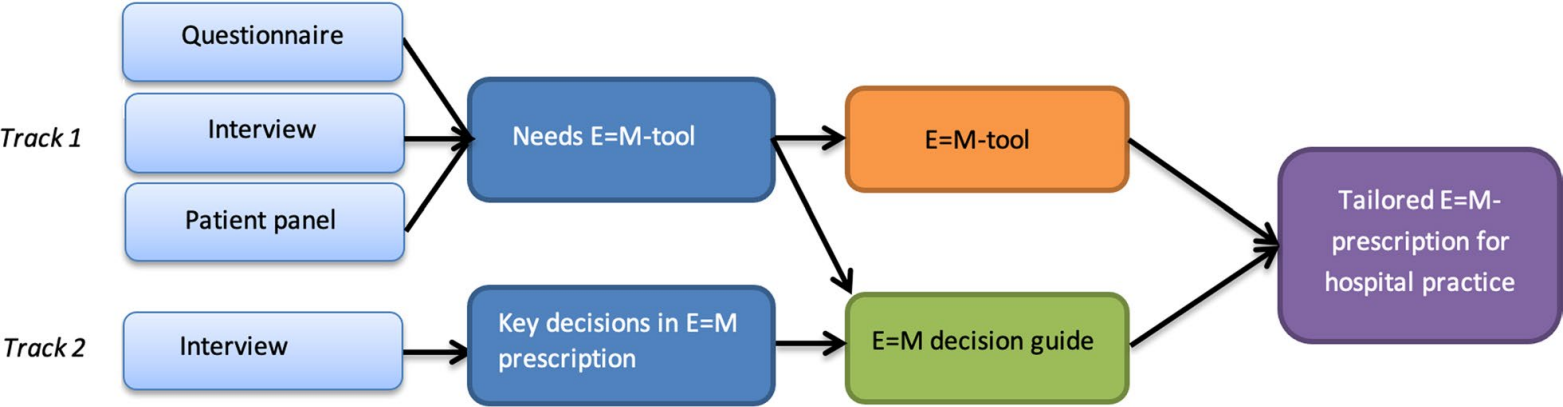
Design to develop a digital E = M-tool and E = M decision guide for the implementation of tailored Exercise is Medicine in hospital care

We used a two-track research design (Fig. 1). Within the *first track* we explored technical needs regarding an E = M-tool with a digital questionnaire, face-to-face interviews and a patient panel. With this information we developed a digital E = M-tool, in close cooperation with stakeholders.

Within the *second track* we applied a qualitative design, to assess key decisions for implementation of E = M in clinical care, by conducting face-to-face interviews. Based on these key decisions, we created an E = M decision guide, in close cooperation with stakeholders.

### Track 1: needs assessment for an E = M-tool

#### Participants

To participate in the needs assessment for an E = M-tool in hospital care, outpatient clinicians from Rehabilitation Medicine and Orthopedics (medical specialists, specialist registrar, physician assistants and nurse practitioners), lifestyle coaches, IT specialists and hospital managers from the departments of Rehabilitation Medicine and Orthopedics of the University Medical Center of Groningen in the Netherlands were recruited via e-mail in November 2018. Lifestyle coaches are (para)medic professionals with an qualification for lifestyle counseling, working within the outpatient clinic in close cooperation with clinicians. IT specialists are experts from the hospital in medical information management and -technology, who are concerned with the EMR.

#### Questionnaire

To obtain needs for an E = M-tool we used a single digital questionnaire distributed among outpatient clinicians from Rehabilitation Medicine and Orthopedics, lifestyle coaches and hospital managers. The questionnaire was based on a commonly used model of the participating IT specialists of the hospital to identify the needs of an application linked to the EMR, including 39 closed-ended and ten open-ended questions, on: the objective of the project, requirements, user objectives, technical aspects, user stories, content of the tool (Additional file 1: Appendix A). Participants were informed by email about the purpose and procedure of the study before they filled out the questionnaire. Finishing and returning the questionnaire by e-mail was connotated as consent. The questionnaire took about 15 min to complete.

#### Interview

Next, individual, semi-structured interviews were conducted to inquire more deeply the needs of an E = M-tool among clinicians, lifestyle coaches and hospital managers, who completed the questionnaire. Interview topics were determined (Table 1), based on the expertise of the researchers, IT specialists of the hospital and implementation experts. The interview addressed the following topics: objectives for using the E = M-tool, tool input, tool output and technical aspects. Interviews took about 30 min, were audio-recorded and conducted by tree post-doctoral researchers (AB, FvN, JN) not involved in patientcare.

**Table 1:** Semi-structured interview guide to explore the needs for an E = M-tool in hospital care

Items	Questions
Objectives for using the E = M-tool	What are objectives for using the E = M-tool?
	Who should be able to use this tool?
	Who should be able to view the information?
	What should be your role in using this tool?
	Should it be possible to protect certain data from certain job profiles?
	Should data be shareable with other departments, healthcare professionals, external parties?
	Do you have any additions?
Input for an E = M-tool	What patient information should be measured?
	Prompts: exercise, motivation, health, other?
	How is the patient information obtained?
	Which decision tree and norm values are used to weigh the input?
	Do you have any additions?
Output of an E = M-tool	What output is generated?
	Prompts: PA benefits, health gain by exercise?
	Are scores compared to norm values?
	For whom is the output?
	Is it just a referral tool, or should also an exercise advice be given?
	How is the PA advice visualized?
	Is the PA advice stored in the EMR?
	Is the PA advice one-off or does it have follow-ups?
	Is the PA advice shared with other paramedics?
	Is feedback given on the patient's progress in the follow-up?
	How do you want to receive the feedback on the patient’s progress?
Technical aspects of an E = M-tool	Does the tool need to be linked to the EMR?
	Should specific patient data be retrievable from the EMR?
	What security requirements must be applied regarding data security of medical information?
	Who should have access to the tool?
	With which providers will be collaborated?
	Should conditional regulations be applied about privacy and data security?

*E* = *M* Exercise is medicine, *PA* physical activity, *EMR* Electronical Medical Record

#### Patient panel

Three patients from Rehabilitation Medicine of the University Medical Centre of Groningen, two female and one male, were recruited by their medical specialists in March 2019 to represent the patient perspective with a patient panel. They were experienced with PA stimulation in medical treatment and were willing to give their opinion on the use of an E = M-tool during consultation.

The patient panel was focused on the use of the E = M-tool. Topics included: tool input and output; PA advice and the PA referral options (Additional file 2: Appendix B). The session took 60 min, was conducted by researcher AB and was audio-recorded and summarized.

#### Analyses

Results were analyzed by a thematic analysis, using an deductive thematic approach. Outcomes of questionnaires were grouped, consensus was evaluated and key findings were determined by three researchers (AB, FvN, JN). They reviewed the outcomes of the interviews and the patient panel and categorized findings, resulting in an overview of needs presented in the results section (Table 4). We applied triangulation of data sources. Outcomes served as the basis for tool development in close collaboration with stakeholders.

#### Stakeholders

At the start of the PIE = M study in September 2018 we installed a broad stakeholder panel linked to both academic hospitals. These 22 stakeholders, others than those who participated in the needs assessment, included: researchers (n = 5), clinicians from Rehabilitation Medicine and Orthopedics [5], lifestyle coaches (n = 2), municipal PA intervention experts (n = 3), implementation experts (n = 2), a patient representative, IT specialists (n = 2) and software providers RoQua®(UMC Groningen) and Klik®(Amsterdam UMC) (n = 2). Software providers built interfaces to link the E = M-tool algorithm to the EMR. The stakeholders reflected on the results of the needs assessment (objectives for using the E = M-tool, tool input, tool output and technical aspects) during one group consultation, and advised on the tool development. Sessions took about 60 min and were conducted under supervision of three researchers (AB, FvN, JN). Then, different stakeholders (n = 7) pre-tested the use of the E = M-tool prototype individually and gave written feedback by mail on the different subsections of the tool: introduction page, dashboard, consent, assessments of patient information, end of the patient questionnaire, data storage, norms and cut-off points, PA advice, referral options, print options, general issues, configuration, clinician account, further suggestions for the tool. Following their findings, content and functionality were discussed and refined resulting in the final version of the E = M-tool.

### Track 2: Key decisions for implementation of E = M

#### Participants

Outpatient clinicians (medical specialists, specialist registrar) and hospital managers participated from the departments of Rehabilitation Medicine and Orthopedics of the University Medical Center of Groningen and of the department of Rehabilitation Medicine of the Amsterdam University Medical Centers in the Netherlands. They were recruited via email in January 2019 to explore key decisions for implementation of E = M. Participants were partly the same as in the needs assessment of *track 1*. The same stakeholders as in *track 1* were involved to reflect on the outcomes during group meetings to develop the E = M-decision guide for hospital care.

#### Interview

Key decisions were explored for implementation of E = M by conducting semi-structured interviews with clinicians, lifestyle coaches and hospital managers. The interview topics were defined guided by the Framework for Innovation within Healthcare Organizations [36] (Table 2). Consensus was reached on the specific interview topics about decision-making for implementation of E = M. For this purpose, a visualization of a tool draft was shown on paper, to support a response. We investigated which steps and accompanying decisions had to be made in order to facilitate the implementation of E = M by clinicians, supported by an E = M-tool. All participants provided written informed consent. The interviews took 60 min, were conducted by three researchers (AB, FvN, JN), audio-recorded and verbatim transcribed.

**Table 2:** Semi-structured interview topics to explore key decisions for implementation of E = M in hospital care

Topics	Items	Questions
E = M implementation	General	How is the implementation of PA advice and referral to PA interventions organized now in your setting?
		How would you like the PA advice and referral to be organized?
		What are your thoughts on the implementation process of E = M in your department?
	PA advice	What should be the content of the PA advice?
		What target groups are eligible for PA advice?
		How should the PA advice be provided?
		What information (incl. cut-off points) should be used in the algorithm to provide a PA the advice?
	PA referral	What are referral options?
		How does a referral work?
	Implementation	Who are the actors in the implementation of E = M?
		What is a suitable timing of E = M in the process of medical treatment?
		What are facilitators of E = M in your specific department?
		What is helpful in the implementation of E = M?
E = M-tool	Tool draft	What is your reaction to the tool draft?
	Content	What should be input for the tool?
		What should be the function of the tool?
		What should be output of the tool?
		What patient groups should be included?
	Implementation	How is the tool used in the work process?
		How should the embedding of an E = M-tool be optimized?

*E* = *M* Exercise is Medicine, *PA* physical activity

#### Analyses

**Fig. 2:**
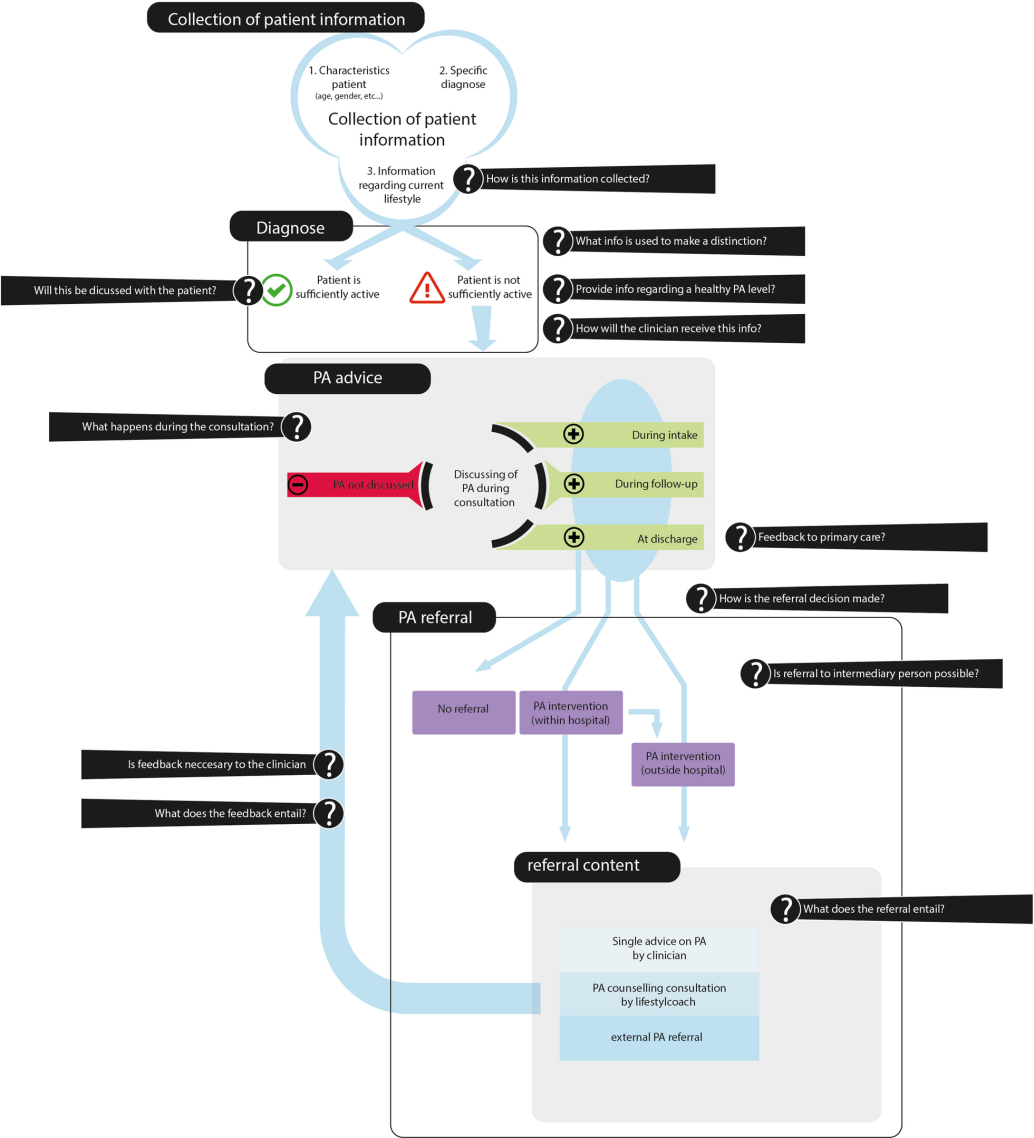
E = M decision guide in the decision-making to facilitate E = M in hospital care

The interview data were analyzed in Atlas.ti by an inductive thematic analysis, by three researchers (AB, FvN, JN), using a semantic approach [37]. The three researchers reviewed the interviews and categorized outcomes into five main points of decision-making. Then, for each point of decision-making, we defined accompanying questions to gain insight into what information is used and provided, what resources and channels are used, based on *track 1* and *2*. Outcomes were reviewed with stakeholders. Two researchers (AB, JN) made the overview presented in de result section (Fig. 2).

## Results

### Track 1: needs for an E = M-tool

Twenty participants completed the questionnaire (12 clinicians, 3 lifestyle coaches, 3 hospital managers, 2 IT specialists), of whom 16 were willing to participate in interviews (10 clinicians, 3 lifestyle coaches, 3 hospital managers). Demographic details of participants are provided in Table 3.

**Table 3:** Characteristics of participants from the needs assessment and analyses of key decisions for implementation of E = M

	Track 1: Needs assessment for an E = M-tool in hospital care		Track 2: Key decisions for implementation of E = M	
	Questionnaire	Interview	Patient panel	Interview
*Gender*				
Male	9	6	1	11
Female	11	10	2	8
*Hospital*				
UMC Groningen	20	16	3	13
Amsterdam UMC	–	–	–	6
*Department*				
Rehabilitation medicine	9	8	3	10
Orthopedics	9	8	–	9
IT	2	–	–	–
*Function*				
Medical specialist	4	3		8
Specialist registrar	5	5		6
Lifestyle coach	3	3		–
Hospital manager	3	3		5
Hospital IT specialists		2		–
Phycisian assistant	1	1		–
Nurse practitioner	2	1		–

*E* = *M* Exercise is Medicine, *UMC* University Medical Cente, *IT* Information Technology

Identified needs in both academic hospitals are described in Table 4. Most mentioned needs were that ‘the tool should identify an inactive lifestyle’, ‘it should generate an individually PA advice’ and ‘a tool should determine which patients are eligible for PA referral’. The tool should be a digital algorithm ‘linked to the patient’s EMR file’ and it should ‘decrease the workload’ in clinicians.

**Table 4:** Identified needs for an E = M-tool in the participating hospital departments, resulting from the needs questionnaire, interviews and patient panel (Track 1)

Items	Results
User objectives	Able to generate a PA advice
	Able to select eligible adult patients for referral to PA interventions
	Usable during consultation by clinicians and patients
	Usable as research data by researchers
	Track the use of the tool among clinicians for research purposes
Input	Patients provide information about activity level, concerning
	Current PA behavior
	BMI
	Motivation to change PA behavior
	Personal characteristics: age, gender, etc
	Diagnosis
	Co-morbidity
	Intoxication
	Health related quality of life
	Use of international PA guidelines
	Use of PA levels/guidelines tailored to diagnosis groups
Output	Tool should generate tailored PA advice on patients’ diagnosis indicate
	The urgency to be more physically active
	Willingness to change
	Need for PA coaching
	Scores are compared to guidelines
	Predict the personal benefits of PA
	PA advice is short, simple and visualized with symbols and color coding
	Generate referral options in/outside hospital
	PA advice is one-off, stored in EMR, printable and handed out to patient
	PA advice is not necessarily shared with paramedics outside hospital
	Feedback is not necessarily given on the patient's progress at follow-up appointments
Technical aspects	A digital tool is required
	Include an algorithm to compare patient’ scores with PA guidelines
	Linked to existing hospitals’ EMR
	Provided by a reliable and safe system
	Data can be retrieved from EMR
	All clinicians, with a treatment relation to the patient from the same hospital have tool access
	Clinicians should be able to add medical information
	Software providers RoQua/Klik should collaborate
	Local privacy and security regulations of medical data are applied
User stories	E = M-tool should make implementation of E = M as easy as possible for all entities
	The tool should show the results and generate advice through the EMR
	The tool should provide individual tailored PA advice
	Patients should provide information for their PA advice
	Researchers should inform patients about the use of the tool and the PA advice
	Researchers should have access to the data for research (after patient consent)

*PA* physical activity, *BMI* Body Mass Index, *EMR* Electronic Medical Record

#### Tool development

Based on the needs assessment of an E = M-tool, an interface within the EMR was developed for the two participating academic hospitals by two commonly used digital application providers. Firstly, results from questionnaire and interview (*track 1*) were used to design the context diagram (Additional file 3: Appendix C), representing all entities that are required to interact with the E = M-tool through defined processes and was designed for IT specialists and RoQua and Klik providers to provide information about the required processes and functionalities. Secondly, the results served to design an algorithm (Table 5) on PA level, BMI, motivation and diagnosis. The self-reported PA measurement used the validated questions of the Scottish Physical Activity Screening Questionnaire (Scot-PASQ) [38]. BMI was assessed using self-reported height and weight. When the PA or BMI did not meet the guidelines or when the patient had a PA-related question, he/she was eligible for referral. Referral means that a patient is referred to a lifestyle coach at the outpatient clinic for a one-time, personal lifestyle advice, with a focus on PA. If the patient needs more guidance, he/she is referred via the lifestyle coach (as PA broker) to an intra- or extramural PA intervention. Patient’s motivation was measured based on the Trans Theoretical Model [39]. The motivation rate did not influence the outcome of the algorithm but was used as indicator for the approach of the conversation to increase patient’s PA. With this algorithm, inactive patients and/or patients with overweight were selected, based on PA or BMI norms. It provided a customized PA-advice (Additional file 4: Appendix D).

**Table 5:** Example of a E = M-tool algorithm

Assessment	Physical activity level (PA)	Body mass index (BMI)	Motivation	Diagnosis
Question	In a normal week, on how many days are you physically active for a total of 30 min or more? (You can think of: walking, cycling in free time or as transport, gardening or sports for at least 10 min in sequence)	What is your height	Rate your motivation to change your PA behavior on a scale from 1 to 10	What is the preliminary primary diagnosis for treatment of this patient?
		What is your weight	Are you willing to be advised over your PA behavior or do you have any PA-related question for your clinician or a lifestyle coach? (Yes/No)	
	Are you physically active for at least 21/2 h (150 min) in a normal week?			
Result	PA ≥ 5 days/week—> advice 1	BMI ≤ 25—> advice 1	1: 1–10	pre-formulated types of diagnosis
			2: Yes/No	
	PA < 5 days/week—> advice 2			
		BMI > 25—> advice 2		
	PA ≥ 150 min/week—> advice 1			
	PA < 150 min/week—> advice 2			
Advice	Patient is sufficiently active	Patient has a healthy weight		pre-formulated benefits of PA per diagnosis type
	Patient is insufficiently active and patient is eligible for referral to a PA expert	Patient is overweight and is eligible for referral to a PA expert		
Note	Answers to this measurement are self-reported by the patient	Calculation BMI = weight/height^2^	Motivation score is used as indicator for the lifestyle advisor to what extent the patient is ready to change	Type of diagnosis (preformulated) is filled in by the clinician
		Answers to this measurement are self-reported by the patient	Even when the patient indicated ‘no’ to this question, he/she still is eligible for PA referral when the patient is insufficiently active or overweighed	

*PA* physical activity, *BMI* Body mass index

### Track 2: key decisions for implementation of E = M

After analyzing nineteen interviews with fourteen clinicians and five hospital managers about decision-making in the implementation of E = M in hospital care (see Table 3), five points of decision-making on the implementation of E = M were distinguished: collection of patient information; diagnosis; consultation; PA advice; PA referral. These parts formed the basis for the E = M decision guide, with the purpose of guiding the tailoring of E = M to a clinical context. The E = M decision guide provides what decisions have to be made in order to facilitate the implementation of E = M in practice. For each point of decision-making, accompanying questions are formulated for tailoring to practice (Fig. 2). It starts with the *collection of patient information* as input for a PA diagnosis. Choices will have to be made about which patient characteristics are included and how this information is collected. Then, it is determined which information is used to *diagnose* sufficiently vs. insufficiently active patients. Choices have to be made about how the practitioner obtains this information to discuss the patient information during the consultation. Then, choices have to be made about the *PA advice:* what information is provided, what is discussed, when and in what way. It should also be considered whether the PA advice is communicated to other practitioners and how. There are several options for a PA referral. The patient does not get a referral because he/she is sufficiently active or not eligible. Or, the patient is referred to the PA intervention option inside the hospital (PA broker/lifestyle coach centrally in the hospital or on the department) or outside the hospital (PA coaching in living environment of the patient). This can be done directly by the clinician or through an intermediary. Finally, a choice has to be made about the content of the PA intervention and whether and how feedback on this is given to the main physician.

### E = M-tool for hospital practice

This decision guide for implementation of E = M was tailored to current hospital departments. It indicates per hospital in this study on each point of decision-making which choices have been made for implementation. Although, comparable choices have been made, there were differences on target group, content of PA advice and referral to an intermediary person within the hospital (Table 6).

**Table 6:** Decisions of E = M-tool prescription tailored to hospital departments in this study

Points of decision-making	Questions for tailoring to practice	Decisions	
		UMC Groningen (Rehabilitation, Orthopedics)	Amsterdam UMC (Rehabilitation)
The collection of patient information	What is the target group?	Patients with complaints on:	Patients diagnosed with
		Shoulder	Multiple scleroris
		Hand	Chronic pain
		Hip	
		Knee	
		Ankle	
		Foot	
	What patient-information assessed?	Personal characteristics	Personal characteristics
		PA	PA
		BMI	BMI
		Motivation	Motivation
		Need to discuss PA with a PA expert	
		Diagnosis	
	How is patient-information collected?	With a digital questionnaire linked to the EMR	With a digital questionnaire linked to the EMR
	How are patients informed and how are login details send out for questionnaires?	Researcher (during pilot)	Medical administration (during pilot and usual care)
		Medical administration (during usual care)	
The diagnosis	How will the clinician receive the patient-information?	In the EMR file	In the EMR file
	What information is used to make a distinction between sufficiently active or not?	ACSM PA norm [13]	ACSM PA norm [13]
	What cut-off points are used in the algorithm?	PA < 150 min	PA < 150 min
		BMI norm > 25	BMI norm > 25
			Motivational cut-off point
	Will the PA advice be discussed with the patient?	Discuss PA advice with clinician/PA expert	Discuss PA advice with clinician
The consultation	How is patient-information obtained for the PA advice?	Automatically generated PA advice visible via the EMR	Automatically generated PA advice visible via the EMR
	Where does it fit in the care process?	During intake and follow-ups	During intake and follow-ups
	What is the content of the PA advice?	Results of assessed patient information	Results of assessed patient information
		Comparison of results with guidelines	Comparison of results with guidelines
		Tailored PA advice	Tailored PA advice
		Diagnosis specific benefits of PA	Conversation suggestions for physicians to motivate patients
		Eligibility for referral	Online referral options
		Possibility to adapt advice to personal circumstances	
		PA referral options in/outside hospital	
The PA advice	What are criteria of the output?	Automatically generated PA advice within the EMR	Automatically generated PA advice within the EMR
		Visualized with colored symbols	Visualized with colored symbols
		Visible in the patient profile of the EMR	Visible in the patient profile of the EMR
		Printed PA advice and handed to the patient, optionally sent by email	Verbally during consultation
		Saved in de EMR	
	What are the PA referral criteria?	ACSM PA norm [13] < 150 min, or:	ACSM PA norm [13] < 150 min, or:
		BMI norm > 25, or:	BMI norm > 25
		Need to discuss PA with a PA expert	
	Is referral to intermediary person possible?	Yes	No
	Is feedback of PA advice/PA intervention given to other medical professionals?	No	No
	Extra information and guidance	Handout with websites and Apps	Verbally websites + KLIK page
The PA referral	Is referral to intermediary person within the hospital possible?	Consultation with lifestyle adviser, physiotherapist or sport consultant	Physiotherapist
	What is the content of the consultation with an internal intermediary?	PA preferences	Current PA behavior
		PA goals	Inform about PA guidelines
		Motivation for PA	External referral options
		Barriers to PA	
		Action plan	
		External referral options	
	What are the external PA referral options outside the hospital?	PA interventions in patient’s vicinity	PA interventions in patient’s vicinity
		Primary care lifestyle interventions	Primary care lifestyle interventions
		Regular referral options, e.g.: physiotherapist	Regular referral options, e.g.: physiotherapist
	Is feedback given to a physician of the PA advice?	No	Yes, to GP with copy of PA advice in a GP letter

*UMC* University Medical Center, *PA* physical activity, *BMI* body mass index, *EMR* Electronic Medical Record, *ACSM* American College of Sports Medicine, *KLIK* Dutch survey-system Kwaliteit van Leven In Kaart, *GP* general practitioner

## Discussion

To support the implementation of E = M in hospital care we provided a supportive E = M-tool for an individual PA advice and referral options, based on specific needs of hospital departments in this study. Additionally, this study developed a guide to E = M decision-making on key decisions for implementation of E = M in hospital care.

### Track 1: E = M-tool in hospital care

The main technical requirements determined in this study for an E = M-tool are that it should be a digital tool, usable during the patient consultation, that it should select eligible adults for referral to PA interventions. In terms of content, most mentioned requirements were that the tool indicates per patient, the urgency to become more physically active, that it reflects the current PA behavior, the motivation to change and the need for coaching. We integrated these requirements in the study by developing a digital tool, which could immediately generate a PA advice during the consultation, taking into account patient characteristics, as*:* current PA level, BMI, diagnosis, motivation and need for advice. It generated an individual PA advice by using an algorithm, based on the norm values of specific patient data. Also the algorithm described the personal benefits of being more physically active and referral options. The value of this information being included in the EMR alerts healthcare professionals to the opportunity to discuss PA with the patient and the need for referral. A more tailored PA prescription should come from a professional in consultation with the patient.

In the tool-algorithm, self-reported PA is assessed using the one-item PASQ [38]. After careful consideration, we chose these questions over the PAVS [40] recommended by ACSM. Because the tool in this study is used to give an indication of PA behavior and to initiate a conversation, it was decided not to measure the intensity per activity. Additionally, end-users in our study estimated a difficulty for patients to indicate perceived intensity of physical activities. They expected an overestimation by patients. The PASQ's questions proved to be valid and met the needs of our users and stakeholders. The E = M tool may also be used for ongoing monitoring during treatment. In our interrelated study it was already indicated by clinicians that continuous monitoring of PA behavior is recommended [28]; If the PA behavior decreases considerably, this may be a sign that the patient is not doing well. When such a tool is used to set up a tailored training program or to measure effects of a medical treatment, PA measurement should include intensity.

As a result of the needs assessment among clinicians it was decided to also include BMI in order to select patients eligible for a PA referral. This obviates that people are not referred who do meet the PA criteria, but overestimate their PA behavior. BMI is assessed using self-reported height and weight. A self-measurement was chosen because of the lack of time during clinical consultations. These are examples of choices balancing between usability and validity. Besides PA and BMI age, gender, medical diagnosis, motivation to change physical activity, and preference to discuss physical activity with their doctor were also included.

In line with the E = M implementation by Kaiser Permanente Southern California, a large healthcare system in the U.S.A, our tool seems appropriate for implementation of E = M, because the systematical assessment of PA levels in patients during consultation. Integrating PA assessments into the EMR is a good method to prompt clinicians to make PA assessment and interventions a priority [23, 25]. This would result in improved PA documentation in patient files and PA referrals [23, 24]. Additionally, the use of EMR linked PA assessments, PA advice and referral is suggested as efficient and effective manner to incorporate E = M in the working process of health professionals in the review of Bowen [33]. Several other studies on the use of EMR-systems or web-based decision aids for patients have also shown positive effects of health tracker systems on patient outcomes and its potential to improve patient’s health [41–43]. We hope we developed an effective tool to increase implementation of E = M, adjusted to the work process of the users. Our E = M tool should be further investigated by pilot testing whether this tool is effective and feasible for daily clinical practice.

### Track 2: E = M decision guide

The implementation of E = M is not only about a digital application, it is about what is needed to arrive at PA advice and referral to PA interventions. Five points of decision-making key were identified from interviews: collection of patient information; the diagnosis; the consultation; the PA advice; and the PA referral. In the use of medical decision-making, the E = M-tool may facilitate clinicians’ E = M referral behavior by providing a formal procedure. Human error and subjective influences from clinicians are reduced. The use of such an E = M procedure can reinforce the clinicians’ self-confidence to engage in shared decision-making regarding lifestyle. Persson et al. [44] indicated that general practitioners expressed a need for procedures and guidelines when it comes to lifestyle advise. Additionally, a cooperation with shared procedures on E = M between health professionals in and outside the hospital may have a positive effect on perceived barriers in healthcare [18, 30, 31].

### E = M-tool for hospital practice

With the decision guide, E = M implementation was defined per hospital and department in this study. However, this study provided different interpretations of E = M, based on different needs on patient population, what patient information was collected, the content of the PA advice, the E = M referral options and the output that was shared with the patient (Table 5). Our E = M-tool development and E = M decision guide should serve as an example, which can be tailored to the specific setting in other healthcare institutions.

### Limitations

Generalizing results to other hospitals should be done with caution. The participating clinical departments may have had a stronger focus on the importance of PA compared to other clinical departments. Both hospitals involved are academic hospitals, which implies that mainly complex care is provided. This may have affected the needs for a supportive E = M-tool and the needs to arrive at the implementation of E = M. Also, because participants participated voluntarily, it may be assumed that participants involved were a positive sample regarding the importance of PA behavior and E = M. However, we do not expect that the needs for an E = M tool and key decisions for implementation of E = M will differ greatly towards other settings and participants.

### Future research

Pilot testing the decision support system for implementation of E = M, including a clinical E = M-tool, would be a next step within research on implementing E = M in clinical care. In order for PA advice and promotion to be embraced by healthcare systems, care providers, patients, health insurance, and technology, E = M needs to be incorporated into the standard care of healthcare institutes in a sustainable way. We should learn, cross nationally, how to adapt successful models to make E = M an efficient workable standard in healthcare.

## Conclusion

This study provides insight into the needs from clinical settings for an E = M-tool and key decision in the implementation of E = M in clinical care. We learned that there are many decisions to be made to facilitate the implementation of E = M tailored to a specific setting. Outcomes may serve as an example for other decision support guides and interventions aimed at E = M implementation.

## Supplementary Information

**Additional file 1**.** Appendix A**. Questionnaire for the needs assessment for developping an E = M-tool.

**Additional file 2**.** Appendix B**. Interview guide patient panel for the needsassessment of developping an E = M tool.

**Additional file 3**.** Appendix C**. suplementary figure 1: Example of a context diagram of the data process of the E = M-tool linked to the EMR in which most important processes (P1-P5) and entities (patient, clinician, researcher) are determined.

**Additional file 4**. **Appendix D**. Example of a costumized PA-advice as output of an E = M-tool linked to the EMR.

## Data Availability

The datasets generated and/or analyzed during the current study are not publicly available due to violation of the individual privacy. Data from questionnaires and transcripts of interviews are only available on request (because of traceability). The e-tools used have been developed in existing software systems (RoQua and KLIK), and are available through these organizations. The e-tool in RoQua requires integration with an electronic medical record (EMR). KLIK is stand-alone software, and is available (open-access) for KLIK users. The datasets generated and analyzed during the current study are available in the Groninger Data Catalogus repository, [https://groningendatacatalogus.nl/menu/groningendatacatalogue/dataexplorer/details/umcg_collections/aaaac6pkomkvd6qwh3dps4yaae].

## Abbreviations

PA: Physical activity
WHO: The World Health Organization
E = M: Exercise is Medicine
EMR: Electronic medical records
PIE = M: Physicians Implement Exercise = Medicine
UMC: University Medical Center
BMI: Body mass index
ACSM: American College of Sports Medicine
KLIK: Dutch survey-system Kwaliteit van Leven In Kaart
