# Supplementary material for: Implementing Exercise = Medicine in routine clinical care; needs for an online tool and key decisions for implementation of Exercise = Medicine within two Dutch academic hospitals
Source: BMC Med Inform Decis Mak. 2022 Sep 22;22:250. doi: 10.1186/s12911-022-01993-5 (PMC9494771; doi:10.1186/s12911-022-01993-5)
Supplement: Supplementary file 1 — Additional file 1. Appendix A. Questionnaire for the needs assessment for developping an E = M-tool. [file 12911_2022_1993_MOESM1_ESM.pdf]

APPENDIX A

Questionnaire needs assessment E=M-tool

Objective of the project

The first objective of the current project is to implement E=M (Exercise is Medicine) in your department in routine care. Second, the project aims to develop a tool that can be used by clinicians of your department and results in a referral to an active lifestyle intervention for your patients. An algorithm will form the basis for an E=M-tool, which will be developed based on your opinion.

Do you have any additions to the objective of this tool?

Requirements

What matters to you when you think about the system of this tool? Below are a few examples. Can you indicate (yes/no) whether these aspects are important to you? And what additions do you have?

- The system must fit in the privacy and security guidelines of the hospital (yes/no)
- The system should match with the current work processes (yes/no
- One system for different departments within the hospital, with the option of data protection and segregation of duties (yes/no)
- Additions:…..

Users objectives

Which *users* should use the tool to perform what *actions* and what *rights* do users have?

Technical aspects

What matters to you when you think about the technical aspects of this tool? Below are a few examples. Do these apply to you (yes/no), and what additions do you have?

- The tool must be able to be used on PC (access via tablet, mobile optional) (yes/no)
- The tool must work in Microsoft and Mac environment (yes/no)
- The tool must be accessible to hospital staff, also outside the hospital domain (yes/no)
- People without a hospital's warrant must have access (yes/no)
- Additions:
    - What do you think is important about the used *system* of this tool?
    - What matters to you when you consider the technical aspects of this tool?

User stories

What should the tool be able to accomplish, in the context of E=M implementation in your department of hospital care? Here we give some examples of what the tool should be able to do. Make the number of your answer BOLD in each example.

	Totally disagree	Disagree	Neutral	Agree	Totally agree
It must be a tool that is built into the EMR	1	2	3	4	5
The patient must be able to view the exercise advice in a shielded personal health environment on the hospital's website	1	2	3	4	5
It must be possible to send the exercise advice to the supervisor of the lifestyle intervention	1	2	3	4	5
I want to be able to create, modify and delete a new account myself	1	2	3	4	5

I want to be able to enter data myself	1	2	3	4	5
I want to be able to see the advice in one storage	1	2	3	4	5
It must be an easy-to-handle system where I have to enter a maximum of ten parameters	1	2	3	4	5
I want to be able to see follow-ups in the system, so whether there is progress in the patient's exercise behaviour	1	2	3	4	5
The tool must immediately generate personalized exercise advice for the patient, which the doctor discusses with the patient	1	2	3	4	5
The tool should only result in a referral to an exercise intervention, without exercise advice for the patient	1	2	3	4	5
The tool is layered, whereby a doctor uses the tool diagnostically and forwards it to an affiliated health professional who provides exercise advice and develops a tailor-made program	1	2	3	4	5
I want to see a list of potential exercise coaches by zip code and different types of exercise interventions offered to which I can refer my patient	1	2	3	4	5
I want to have a good overview of the correspondence and back-and-forth about a patient with regard to his lifestyle advice, such as the frequency and content of emails or conversations	1	2	3	4	5
I want an easy link with the mail system	1	2	3	4	5
I want to be able to adjust new exercise interventions in the tool	1	2	3	4	5
I want to be able to adjust the distance to sports facilities in the tool	1	2	3	4	5
I want to answer questions from one central point/address, even though different employees are working on it behind the scenes	1	2	3	4	5
I want to have a complete overview of the status of the active lifestyle advice (of handling the advice: advice registered; being worked on, referred)	1	2	3	4	5
I want to be able to mark/flag account and issues, as a warning that an action hasn't been done yet	1	2	3	4	5
I want to be able to print the advice/referral in a handy format and in a good layout	1	2	3	4	5
I want to be able to define tasks and 'to do's' with notification for action	1	2	3	4	5
It must be usable to other service organizations (e.g. GP, community team, lifestyle coach, personal health environment on the hospital’s website), so that issues can be followed	1	2	3	4	5
Additional user stories:..					

Here you can indicate which functionalities the tool should have in your opinion. You can indicate the priority via MUST, SHOULD, COULD.

MUST (this functionality the tool must have, otherwise it will not be usable):
SHOULD (this functionality the tool should have, to really make sense):

COULD (These are the things that would be nice if the tool could do that, but can also be done later):

Content of the tool
What matters to you when you think about the information collected by this tool? Below are a few examples. Make your answer BOLD for each example.
And, what additions do you have? The following information must be collected by the tool:

Data	Measurement	Answer
Sex	m/f	Yes/no
Age	In ages	Yes/no
Patient's current movement behaviour	Min/day	Yes/no
Patient's physical history	Min/day	Yes/no
Patient motivation to exercise more	Stage of change	Yes/no
Patient's physical health	.. (total score?)	Yes/no
Patient's mental health	.. (total score?)	Yes/no
Patient preference		Yes/no
The patient's social environment		Yes/no
Physical living environment of the patient		Yes/no
What do you think are important predictors of exercise behaviour in (your) patients?		

= end of questionnaire=

Thank you for completing this document. Could you email this completed document to xxx. We will discuss this further with you during in an interview.
