# Supplementary material for: Implementing Exercise = Medicine in routine clinical care; needs for an online tool and key decisions for implementation of Exercise = Medicine within two Dutch academic hospitals
Source: BMC Med Inform Decis Mak. 2022 Sep 22;22:250. doi: 10.1186/s12911-022-01993-5 (PMC9494771; doi:10.1186/s12911-022-01993-5)
Supplement: Supplementary file 2 — Additional file 2. Appendix B. Interview guide patient panel for the needsassessment of developping an E = M tool. [file 12911_2022_1993_MOESM2_ESM.pdf]

APPENDIX B

Interview guide patient panel

**Domain**	**Questions (prompts)**
Discussing E=M	- What is your opinion about discussing exercise with your clinician during the consultation?<br>- Should clinicians deliver an exercise advice by default?<br>- What patient groups are eligible for exercise advices?<br>- Should a clinician refer to exercise experts? (*prompts: inside/outside the hospital)*
Input	- When should a questionnaire be completed? *(prompts: before/during consultation)*<br>- How would you prefer to complete this questionnaire? *(prompts: by email, EMR portal, by mail, in the waiting room, etc.)*<br><br>*Show questionnaire draft*<br><br>- What do you think of these questions? *(prompts: understandable, relevant questions, amount of questions)*<br>- Do you think patients will answer these questions truthfully?<br>- What if the results of the questionnaire were shared immediately with the clinician? (*prompts: see the result directly after completing the questionnaire, determine whether other professionals can see it, print it and take it with you*)
Output	- What are your expectations of an advice conversation about exercise? *(Prompts: benefits of exercise, standards, diagnosis of specific guidelines, local range of exercise, advice on BMI, addressing motivation to change, guidance on exercise)*<br><br>*Show draft of the PA advice*<br><br>- What is your opinion about these topics to discussed during consultation?<br>- What is important about the layout of this PA advice?
