# Supplementary material for: Implementing Exercise = Medicine in routine clinical care; needs for an online tool and key decisions for implementation of Exercise = Medicine within two Dutch academic hospitals
Source: BMC Med Inform Decis Mak. 2022 Sep 22;22:250. doi: 10.1186/s12911-022-01993-5 (PMC9494771; doi:10.1186/s12911-022-01993-5)
Supplement: Supplementary file 3 — Additional file 3. Appendix C. suplementary figure 1: Example of a context diagram of the data process of the E = M-tool linked to the EMR in which most important processes (P1-P5) and entities (patient, clinician, researcher) are determined. [file 12911_2022_1993_MOESM3_ESM.pdf]

APPENDIX C

Context diagram

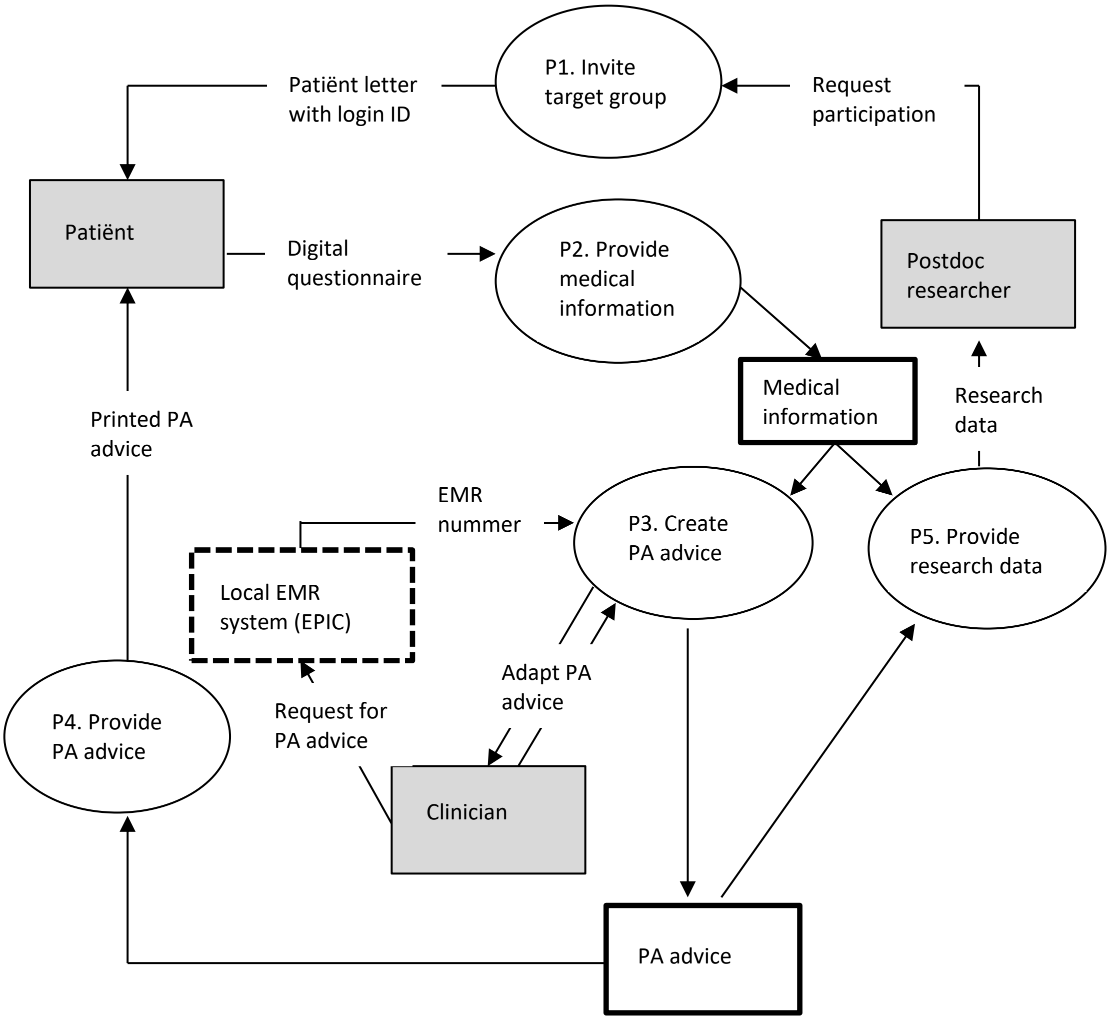

Supplementary figure 1: Example of a context diagram of the data process of the E=M-tool linked to the EMR in which most important processes (P1-P5) and entities (patient, clinician, researcher) are determined.
