# Supplementary material for: Implementing Exercise = Medicine in routine clinical care; needs for an online tool and key decisions for implementation of Exercise = Medicine within two Dutch academic hospitals
Source: BMC Med Inform Decis Mak. 2022 Sep 22;22:250. doi: 10.1186/s12911-022-01993-5 (PMC9494771; doi:10.1186/s12911-022-01993-5)
Supplement: Supplementary file 4 — Additional file 4. Appendix D. Example of a costumized PA-advice as output of an E = M-tool linked to the EMR. [file 12911_2022_1993_MOESM4_ESM.pdf]

Exercise

You indicated that in a normal week you exercise less than 5 days for 30 minutes or more.

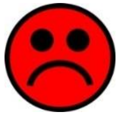

You indicated that you exercise less than 2.5 hours (i.e. 150 minutes) in a normal week.

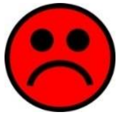

BMI

Your BMI is calculated **27,7**

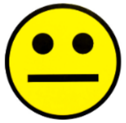

Motivation

Rate your motivation to change your exercise with a 7 (between 1-10)

You indicated that you would like to discuss your exercise with a lifestyle adviser

Diagnosis

Your preliminary diagnosis is: “diabetic feet”

Advice

You are eligible for an exercise referral. A lifestyle advisor will discuss your exercise advice further with you.

In short: you should exercise more per week. We recommend light intensive activities because of the diagnosis of ‘diabetic feet’.

The advantages of exercising more in diabetic feet are:

- Your foot muscles will become stronger and your weight will decrease, which will put less strain on your feet
- Your gait and blood flow will improve.
- You get more flexible muscles, which has a positive effect on your feet and other joints
- You are less likely to get foot injuries
- You will have better carbohydrate absorption by improving insulin sensitivity
- Strong muscles provide more support, which can reduce pain

A lifestyle adviser can further discuss suitable exercise activities with you.

Always use common sense when exercising. If you doubt about the advice, consult your doctor.
